# The Recent Death under Nitrous Oxid Gas

**Published:** 1900-12-15

**Authors:** Dudley W. Buxton

**Affiliations:** M.R.C.P.; Administrator of and Teacher of Anesthetics in University College Hospital, and Senior Anesthesist in the Dental Hospital of London


### THE RECENT DEATH UNDER NITROUS OXID GAS.

BY DUDLEY W. BUXTON, M.D., B.S., M.R.C.P.,

Administrator of and Teacher of Anesthetics in University College Hospital, and Senior Anesthetist in the Dental Hospital of London.

In a paper read (see *Lancet*, June 16, 1900.) before the Harveian Society of London, on May 17th, I drew attention to the peculiar dangers of anesthetics in cases of angina Ludovici and kindred conditions. These conditions are capable of a general anatomical description, and as the case to be referred to in the sequel is a striking instance of death arising through their agency, it may be well to devote some space to their consideration.

Individuals differ widely in the anatomical conformation of what in common parlance is called "the neck." The short or bullock necked persons are recognized as a type, and present in its simplest form the class of cases in which danger from anesthetics may be expected.

The neck structures are less pliant, the head movements less free in these persons. When an attempt is made to turn the head to the side it is resisted by the muscles passing from the trunk to the head. The space between the walls of the faucial opening is usually lessened, and the structures forming it are commonly thick and fleshy. Under nitrous oxid gas the tongue and faucial pillars become engorged. The actual space available for respiration is thus further rendered less, and the dyspnœa increased. A very common associated difficulty in these cases arises from the immobility, or perhaps one should say, the lessened freedom of mobility of the jaws. As the congestion of the soft parts increases an almost spastic closure of the jaws takes place. When, as is so often the case, nasal respiration is impeded by old thickening at the posterior extremities of the nares, by a spur or deflected septum, the perveniousness of the upper airways is still more interfered with and the dyspnœa becomes intensified.

In pathological conditions the impediments arising as a result of thickening of the tissues about the upper air passages are increased, and the incident dangers *sub anesthesia* exaggerated. To understand such conditions it is necessary to refer to the arrangement of the deep cervical fascia. Speaking broadly, this fascia may be said to surround the neck (the superficial layer) and to send inwards to the deeper structures processes which ensheath the muscles and become attached to the vertebræ behind, the posterior surface of the clavicle and inner side of the first rib in front. Other processes pass to the lower jaw, the zygoma, the sternum and muscles in relation with the thyroid cartilage and trachea.

Under normal conditions these fascial septa support, but when inflammation exists they tend to bind, and render the neck structures unduly rigid. It is not uncommon occasionally in cases of lymphadenitis and perilymphaden to meet with extreme rigidity of all the structures adjacent to the upper air passages, leading to difficulties during anesthesia; but the most pronounced thickening occurs in suppuration involving the structures about the lower jaw. The outward appearances in these cases do not always give evidence upon superficial examination to the degree of involvement of the subjacent structures. As pointed out by Ludwig, the suppuration runs along the divisions of the fascia and produces very intense œdema of the tissues about the laryngeal and faucial openings. Œdema of the glottis forms the culminating point of this, involving the greatest danger to life. It is hardly needful to do more than mention that goitres, and especially the "plunging" tumors, constitute another cause of pressure leading to stenosis of the airways. In thickening of the structures of the neck, caused by suppuration, we meet with enlargement of the tonsils, tendency to secretion of thick, glairy mucus, thickening of the structures about the air passages, causing pressure upon them and œdema, which may involve the mucous membrane of the trachea, as well as that of the epiglottis and adjacent structures. The narrowing of the airway thus induced causes dyspnœa, which is more especially inspiratory although expiration is also affected.

In this condition if the patient is given nitrous oxid gas, the result must be, unless means are taken to avoid it, that the venosity of the already carbonized blood is increased, the circulation in the tongue and faucial structures is further interfered with, and the tongue becomes swollen, and this hampers respiration still more. Under such circumstances the liability to dangerous complications is very much increased.

The case of death under nitrous oxid gas reported as having occurred at the Great Northern Hospital, unfortunately only too clearly exemplifies these statements.

In the evidence given before the Coroner's Court, the following facts transpired:

The patient, James Gibbs, a laborer, aged thirty-six, was seen at the Great Northern Hospital, and was suffering from a large abscess involving the tissues on the left side between the submaxillary gland and the ear, but extending also beyond the middle line to the right side. The left tonsil was considerably swollen. The man, although advised to enter the hospital as an in-patient, declined, and had, therefore, as the condition was urgent, to be treated as an out patient. The man was seen at 12 noon, and the operation was undertaken at 4:15 P. M. on the same day. He was said to drink at times, but no evidence exists to give this statement any direct bearing upon the case. No statement is made as to whether or not the man spent the four hours between noon and 4 P. M. in or out of the hospital, but presumably it was outside the institution, and so no special preparation was made. It is common to ignore the necessity of preparation for "gas;" but in surgical cases of gravity, such as the one under consideration, it is wise to make a careful preparation of the patient as regards food, alcohol, etc., even when gas is used without ether or chloroform.

The patient was given nitrous oxid gas by one of the hospital residents, and the following is the description given of the procedure: An "ordinary gas apparatus" was used, fitted with valves. "First pure air, then a few respirations of air and gas, then pure gas" was inhaled. "One bag (? full) and a quarter was given in all." A statement made by the operator, another resident, affirms that "at no time was the patient breathing in and out of the bag." It is difficult to understand that a little over two gallons of nitrous oxid gas would induce complete anesthesia in a man of the laboring class, unless the most complete to-and-fro breathing into the bag was adopted, and a large proportion of carbonic dioxid thus brought into adjuvant action with the nitrous oxid. Probably one or the other statements is open to correction, or, at least, modification.

The man is said to have taken the gas well, by which we may assume cyanosis, stertor and marked dyspnœa were not early phenomena in the case. When ocular reflex was still present ("not entirely gone" ? sluggish) the face piece was removed, and an incision into the abscess was made. The pulse was regular, the respirations were deep and regular, and the pupils of moderate size, and conjunctival reflex was present. The breathing then stopped, "after the excited stage was over." It is not clear what degree of narcosis is referred to "as the excited stage," possibly the appearance of jactitation. The cessation of breathing was accompanied by the appearance of cyanosis. Respiration appears never to have re-appeared after its cessation. As artificial respiration failed to ventilate the lungs, tracheotomy was performed. The necrospsy showed that there was much œdema of the uvula and faucial tissues, also of the epiglottis and upper part of the larynx. The left heart was empty, and both lungs much congested. It is a remarkable feature of this case that no dyspnœa is recorded as having occurred during the induction of an anesthesia. As a rule, not only is dyspnœa present but cyanosis soon appears, and these symptoms of impending asphyxia warn the anesthetist to desist from the use of the anesthetic.

That death was due to œdema glottidis is evident, but how far the giving of nitrous oxid gas accelerated the result must remain an open question. Possibly there may be two opinions. There is no doubt, however, that this particular anesthetic is not the best one for persons with œdema involving their air passages. If given at all, it should certainly be given largely diluted with oxygen. The after treatment of the case also illustrates the fact that no treatment short of opening the trachea offers any chance of success. The apnœa is due to no central failure of nervous control, but simply to mechanical obstruction, and nothing less than the removal of that obstruction can be of the slightest value.

Then, as to the question, Does this case in any way shake the belief that if properly given, nitrous oxid gas is practically a perfectly safe anesthetic? I think not. The selection of the anesthetic in this case was unfortunate, but nothing occurred but what might have been expected, and what possibly might have arisen if no anesthetic had been employed.

In all such cases the risks of anesthesia, even in the most practiced hands are increased, but such risks have reference to the individual and his condition, and not to the anesthetic as such. In fine, one may say that like most, if not all the preceding cases of death under nitrous oxid, the fatality in question simply shows that gas as gas possesses few dangers, and these are those arising from mechanical interference with respiration. These are now well known and can be guarded against or avoided altogether. It also emphasizes the fact that even so safe a body needs care and a judicious decision as to its applicability in any particular case.—*Dental Record.*

